# Age and Age-Related Diseases: Role of Inflammation Triggers and Cytokines

**DOI:** 10.3389/fimmu.2018.00586

**Published:** 2018-04-09

**Authors:** Irene Maeve Rea, David S. Gibson, Victoria McGilligan, Susan E. McNerlan, H. Denis Alexander, Owen A. Ross

**Affiliations:** ^1^School of Medicine, Dentistry and Biomedical Science, Queens University Belfast, Belfast, United Kingdom; ^2^Northern Ireland Centre for Stratified Medicine, Biomedical Sciences Research Institute, University of Ulster, C-TRIC Building, Altnagelvin Area Hospital, Londonderry, United Kingdom; ^3^Care of Elderly Medicine, Belfast Health and Social Care Trust, Belfast, United Kingdom; ^4^Regional Genetics Service, Belfast Health and Social Care Trust, Belfast, United Kingdom; ^5^Department of Neuroscience, Mayo Clinic, Jacksonville, FL, United States; ^6^Department of Clinical Genomics, Mayo Clinic, Jacksonville, FL, United States; ^7^School of Medicine and Medical Science, University College Dublin, Dublin, Ireland

**Keywords:** aging, age-related diseases, inflamm-aging, redox, SASP, autophagy, cytokine dysregulation, inflammation resolution

## Abstract

Cytokine dysregulation is believed to play a key role in the remodeling of the immune system at older age, with evidence pointing to an inability to fine-control systemic inflammation, which seems to be a marker of unsuccessful aging. This reshaping of cytokine expression pattern, with a progressive tendency toward a pro-inflammatory phenotype has been called “inflamm-aging.” Despite research there is no clear understanding about the causes of “inflamm-aging” that underpin most major age-related diseases, including atherosclerosis, diabetes, Alzheimer’s disease, rheumatoid arthritis, cancer, and aging itself. While inflammation is part of the normal repair response for healing, and essential in keeping us safe from bacterial and viral infections and noxious environmental agents, not all inflammation is good. When inflammation becomes prolonged and persists, it can become damaging and destructive. Several common molecular pathways have been identified that are associated with both aging and low-grade inflammation. The age-related change in redox balance, the increase in age-related senescent cells, the senescence-associated secretory phenotype (SASP) and the decline in effective autophagy that can trigger the inflammasome, suggest that it may be possible to delay age-related diseases and aging itself by suppressing pro-inflammatory molecular mechanisms or improving the timely resolution of inflammation. Conversely there may be learning from molecular or genetic pathways from long-lived cohorts who exemplify good quality aging. Here, we will discuss some of the current ideas and highlight molecular pathways that appear to contribute to the immune imbalance and the cytokine dysregulation, which is associated with “inflammageing” or parainflammation. Evidence of these findings will be drawn from research in cardiovascular disease, cancer, neurological inflammation and rheumatoid arthritis.

## Introduction

The inflammatory response must be tightly regulated to ensure effective immune protection. It is a dynamic network that is continuously remodeling throughout each person’s life as a result of the interaction between our genes, lifestyles, and environments (1–3). Infections and tissue damage from the external environment and our personal internal response to stress can act as triggers to initiate the inflammatory defense response. While inflammation is part of the normal repair response for healing, and essential in keeping us safe from bacterial and viral infections and noxious environmental agents, not all inflammation is good. When inflammation becomes prolonged and persists, it can become damaging and destructive (4). It is essential that inflammation is tailored to the initiating stress and resolves in a timely and controlled way, to avoid pathology associated with chronicity.

The cytokine network is a highly complex system of immune molecular messengers, with multiple layers of activation and control mediated through soluble receptors, receptor antagonists, diverse serum mediators, as well as gene polymorphisms (5). Proteomic methods measuring cytokine production and expression have demonstrated further layers of complexity and control in cytokine production and expression involving long coding RNAs, siRNAs, and miRNAs, which make for challenging interpretation of cytokine production and control in the inflammatory process (6). Many cytokines are able to act in more than one-way or paradoxically at different times and many act in feedback loops with the ability to auto-control their own production (7). Cytokine expression is also influenced by local cellular microenvironments, suggesting that multiple pathways exist to achieve homeostatic immunologic control and effectiveness, or to conversely accentuate chronic immune activation. However, what seems clear is that mirroring other body systems, the homeostatic control, titration, and modulation of immune responsiveness becomes more fragile and less tightly focused with increasing age. This loosening of the cytokine balance between the pro-inflammatory and anti-inflammatory control or resolving mechanisms, or inflamm-aging (8, 9), is a characteristic feature of both aging and aging-related diseases. This kind of inflammation is similar to that originally described as “parainflammation” by Medzhitov (10).

Today there is increasing recognition that inflammation is a common molecular pathway that underlies in part, the pathogenesis of diverse human diseases ranging from infection, to immune-mediated disorders, cardiovascular pathology, diabetes, metabolic syndrome, neurodegeneration, and cancer, to aging itself (4, 11, 12). Although there is no exact understanding about the causes of “inflamm-aging”, a common finding seems to involve a dysregulation of the cytokine network and its homeostasis. Several common molecular pathways have been identified that seem to be associated with both aging and low-grade inflammation. Excess oxidative stress and DNA damage trigger the inflammasome, stimulating NF-κB and the IL-1β-mediated inflammatory cascade. Autophagy, the cell machinery process that removes damaged proteins and large aggregates, is also slowed up at older age and in age-related disease, causing damaged material to accumulate and reduce cellular efficiency. Senescent cells increase with age and in age-related diseases, and the associated secretome or senescence-associated secretory phenotype (SASP) produces a self-perpetuating intracellular signaling loop and inflammatory cascade involving the NF-κB, IL-1α, TGF-β, IL-6 pathway that participates in the pro-inflammatory milieu. The molecular processes that damp down inflammation include the resolvin family of bioactive molecules, which have been much less evaluated in aging or age-related disease, but are important participants in effective and timely inflammation resolution.

Here, we will discuss some of the current ideas and highlight molecular pathways that appear to contribute to the immune imbalance and the cytokine dysregulation, which is associated with “inflamm-aging” or parainflammation. Evidence of these findings will be drawn from research in several age-related diseases, including cardiovascular and neurodegenerative disease, rheumatoid arthritis (RA), and cancers.

## The Inflammation Pathway to Resolution

**Figure 1:**
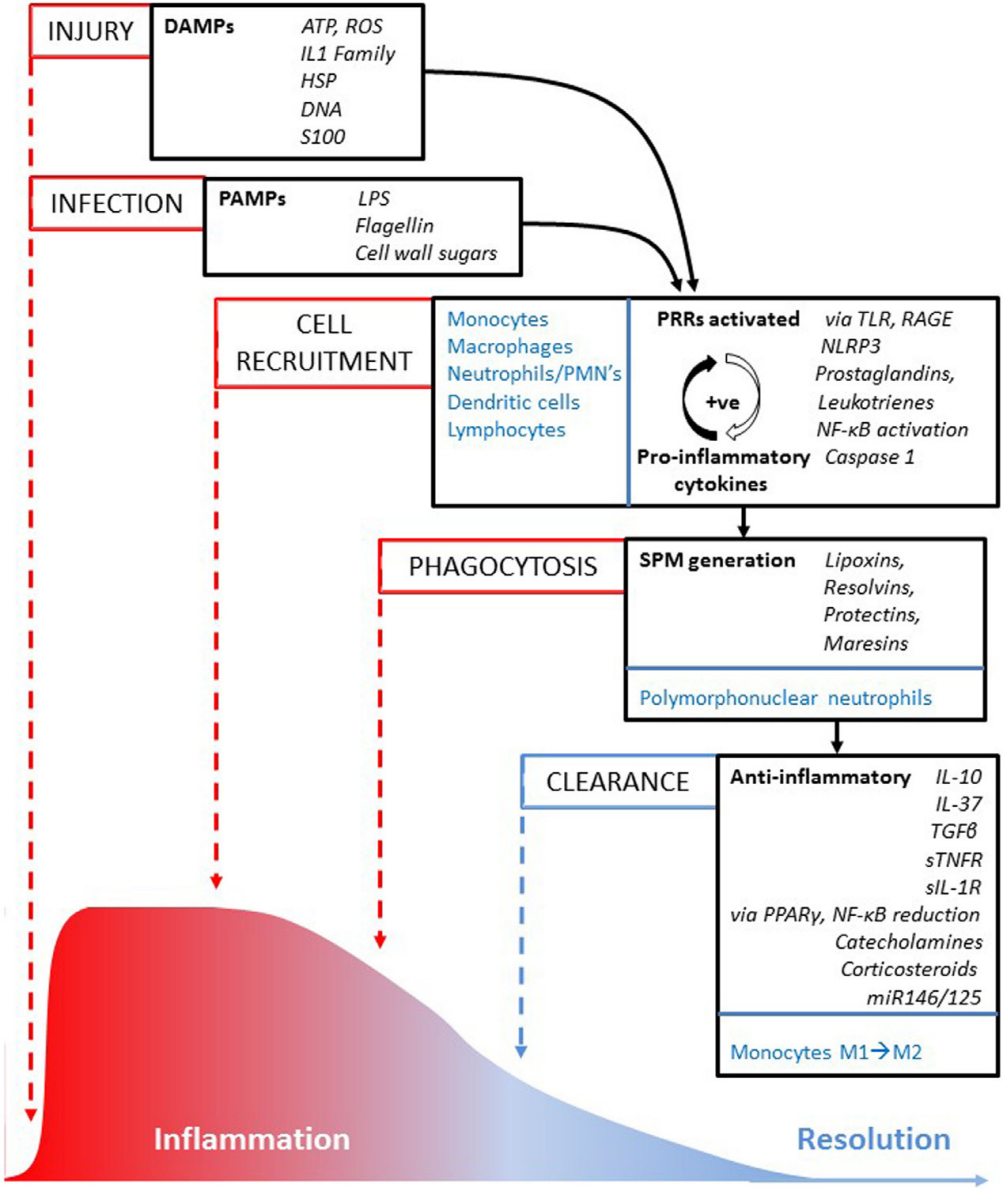
Inflammation pathway to resolution. An illustration of the sequence of key processes (in capitalized text), cells and molecules involved in reaction to injury or infection, and how the inflammatory episode is resolved over time (from left to right). Cells from the innate and adaptive immune system that are involved in cell recruitment, phagocytosis, and clearance processes are highlighted in blue text; key molecules are in italic text.

Inflammation is classically induced when innate cells detect infection or tissue injury. The pattern-recognition receptors (PRRs) on immune cells sense “danger” from protein-associated molecular patterns (PAMPs) associated with pathogens, or from danger-associated molecular patterns (DAMPs) triggered by a wide range of host-derived endogenous stress signals. DAMPs are molecules, such as ATP, the cytokine IL-1α, uric acid, and some cytoplasmic and nuclear proteins, which are released from damaged cells during necrosis and contribute to sterile inflammation (Figure 1). There have been suggestions that the extended IL-1 cytokine family (IL-1α, IL-1β, IL-18, IL-33, IL-36α, IL-36β, and IL-36γ) might also act as DAMPs and stimulate necrosis-initiated sterile inflammation, as well as amplify inflammation in response to infection-associated tissue injury (13).

**Figure 2:**
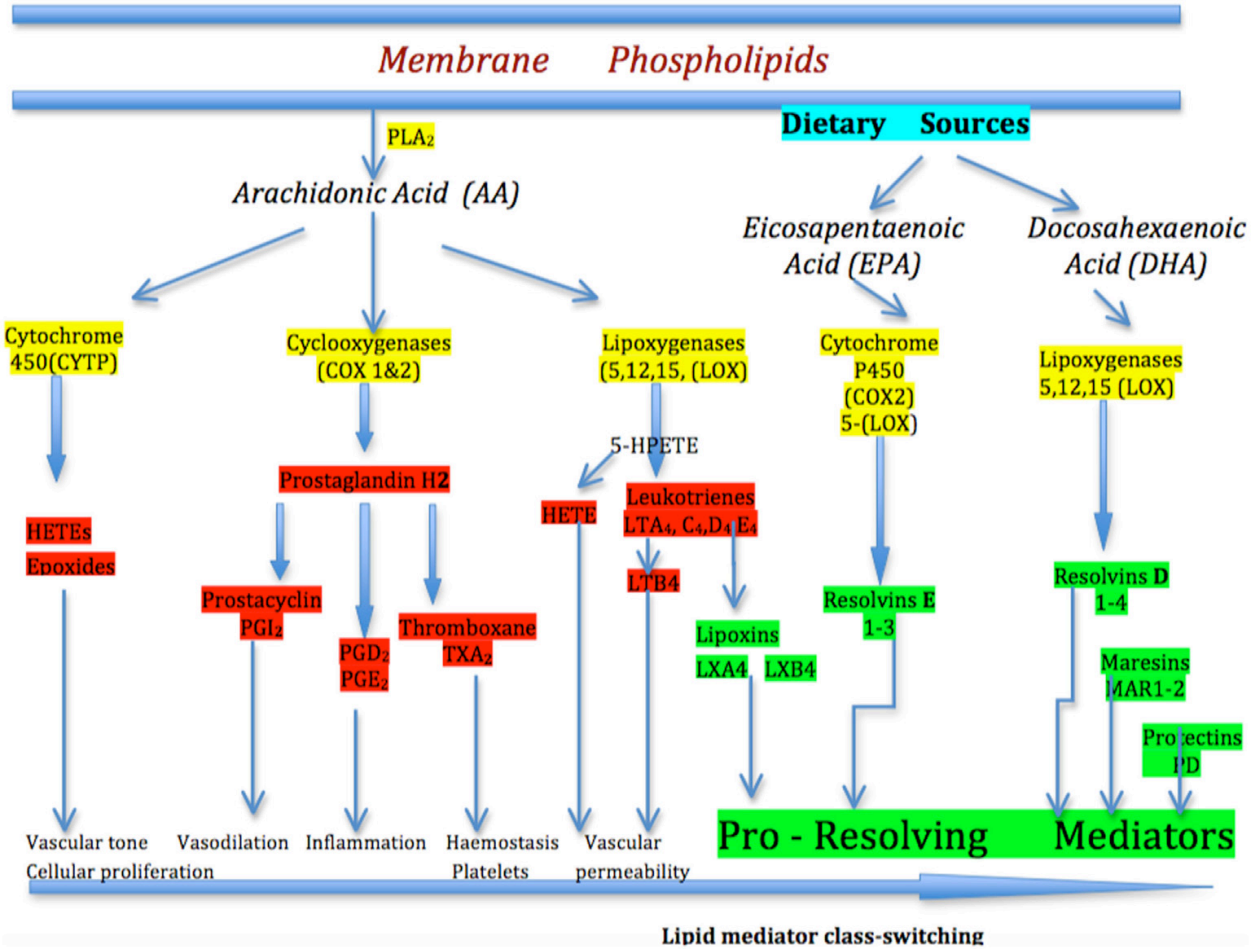
The arachidonic acid (AA) pathway of inflammation mediators. In the simplified pathway for the eicosanoid metabolic pathway, AA is released from membrane stores by phospholipase 2 (PLA_2_). AA is metabolized to biological mediators by three enzymatic pathways: cyclooxygenase, lipoxygenase, and cytochrome P450. Each pathway contains enzyme-specific steps that result in a wide variety of bioactive compounds that drive the pro-inflammatory (prostaglandins) response. After lipid mediator class-switching at the height of inflammation, the pro-resolving mediators-lipoxins begin to drive inflammation resolution. Eicosapentaenoic acid and docosahexaenoic acid-derived from dietary sources produce the E-series of resolvins and D-series of resolvins, maresins, and protectins, respectively, which are important pro-resolving mediators in progressing the resolution of inflammation.

Members of the toll-like receptor (TLR) family are the major PRRs. They are expressed on monocytes, macrophages, neutrophils, and dendritic cells, and on some lymphocytes and they respond rapidly to the “danger” response. The cyclooxygenase (COX) and 5-lipoxygenase (5-LOX) pathways of arachidonic acid (AA) metabolism (14, 15) produce highly pro-inflammatory lipid mediators responsible for the classical signs of inflammation—redness, heat, pain, swelling, and loss of function, with the aim of removing the injurious and noxious stimuli. A third pathway involves the cytochrome 450 pathway of AA metabolism and P450 epoxygenases and hydroxylases that produce both vasoconstrictor and vasodilatory effects in blood vessels and other tissues (Figure 2). The reactive biolipid molecules synthesized from AA are; the prostanoids—prostaglandins (PGs), prostacyclins, and thromboxanes produced by the action of COX 1 and 2 (COX 1 and 2); the leukotrienes (LTs), hydroxyeicosatetraenoids (HETEs), and lipoxins (LXs) produced by the action of the 5-, 12-, and 15-lypooxygenase (5/12/15-LOX) enzymes and; the P450 epoxygenase generates HETEs and depoxyeicosatrienoids (epoxides) (16). PGs act to amplify the inflammatory response through enhancing the inflammatory cytokine cascade, upregulating the innate response to DAMPs and PAMPs, activating subsets of T helper cells, recruiting macrophages associated with chronic inflammation, and increasing cytokine expression from cytokine inflammatory genes. Additional factors, such as histamine, pro-inflammatory cytokines, and chemokines amplify the response further and make the vascular endothelium increasingly leaky. The increase in vascular permeability combined with the expression of cellular adhesion molecules (i.e., selectins and integrins) allows neutrophils, the first responders, to transmigrate across post-capillary venules to the sites of injury or microbial invasion. Together this increases polymorphonuclear (PMN) neutrophil chemotaxis and allows PMNs to transmigrate along chemotactic gradients in order to maximize phagocytosis and killing of pathogens, and deal with the “danger” signal effectively.

As the acute inflammatory cascade develops to manage the “danger” signal, it is essential that a controlled resolution commences, so that immune homeostasis returns in an organized manner. If the inflammatory response does not shut down in a timely way, the inflammation cascade becomes chronic and smoldering. Lipid mediators derived from polyunsaturated fatty acids are now recognized to orchestrate the resolution of inflammation (17). At the peak of inflammation, the eicosanoids that initiated the inflammation undergo a class-switch so that they become the molecules that activate resolution, demonstrable through the clinical signs of removal of symptoms, relief of pain, restoration of function, regeneration of damaged tissues, and return to health. The so-called specialized pro-resolving mediators (SPMs) are key to resolving inflammation and include lipoxins derived from the 5-LOX arm of the AA pathway; the E-group of resolvins derived from dietary-derived eicosapentaenoic acid (EPA); the D-group of resolvins from dietary–derived docosahexaenoic acid (DHA); and protectins (PD), and maresins (MaR) (17–19) (Figure 2). The lipid class-switch starts early in inflammation and is initiated by lipoxins LXA4 and LXB4, and considered to be produced by platelets when they begin to aggregate with PMNs at the sites of inflammation (18).

After class-switching of the lipid molecules has occurred, SPMs are produced. Pro-resolving monocyte-derived macrophages begin to clear PMNs from the site of injury by a process called efferocytosis that removes apoptotic neutrophils, microbes, and necrotic debris. As resolution progresses, monocytes and macrophages, change from a pro-inflammatory (M1) to a pro-resolving phenotype (M2) by genetic and epigenetic reprogramming (20–22). Recent investigations suggest that SPMs, particularly the D-series resolvins (resolving D1 and resolving D2) and MaR 1 modulate adaptive immune responses in human peripheral blood lymphocytes. These lipid mediators reduce cytokine production by activated CD8+ T cells and CD4+ T helper 1 (TH1) and TH 17 cells, but do no modulate T cell inhibitory receptors or reduce their ability to proliferate (23, 24). Other reports show an increase in plasma cell differentiation and antibody production that supports the involvement of SPMs in the humoral response during late stages of inflammation and pathogen clearance (25). The anti-inflammatory cytokines interleukin 10 (IL-10), and IL-37 a member of the IL-1 family, together with TGF-β that is released from monocytes and platelets, are important contributors to damping down the inflammation. The soluble receptors, TNFR and IL-1 receptor (IL-1R) also limit inflammation in acting as decoy receptors, by binding to and neutralizing their respective cytokines, and inhibiting the biological activity. Additional anti-inflammatory mechanisms, include stress hormones, particularly corticosteroids and catecholamines and negative regulators, such as microRNAs—MiR-146 and MiR-125 (26).

The local environment and context also play an important role in the production and function of SPMs, which have both autocrine and paracrine actions. Inflammation resolution is likely to depend on prompt class-switching to pro-resolving lipid mediators, effective apoptosis, and efferocytic clearance of inflammatory cells and debris, timely damping down of pro-inflammatory signals and integrated repair of collateral damage. An imbalance between pro-inflammatory and pro-resolving mediators has been linked to a number of chronic inflammatory diseases (27).

In normal inflammation SPMs do not compromise host immune competence with examples of pro-resolving mediators increasing survival from infections in mouse models (28, 29). The common mechanism by which this occurs appears to be through suppression of the NF-κB activation in a partly PPAR-γ-dependent manner, with associated downstream signaling and alteration in transcriptomics pathways (30, 31). A maresin mediator has been shown to have potent anti-inflammatory and pro-resolving actions in a model of colitis, and attenuated inflammation in vascular smooth muscle and endothelial cells (32, 33). In human studies, the role of SPMs are being explored in chronic inflammatory diseases, such as RA (34), atherosclerosis (27), and cancer (35). In Alzheimer’s disease, several SPMs promoted neuronal survival and β-amyloid uptake by microglia in “*in vitro”* models in Alzheimer’s disease (36, 37). However, little is known about the pro-resolving mediators in aging itself. Studies are needed to assess whether pro-resolving molecules, such as E and D-resolvins, and maresins decrease or are less effective in damping down inflammation with increasing age and whether they could contribute to the pro-inflammatory phenotype associated with aging. Already synthetic analogs are in process of development, and so the design of pharmacological mimetics of naturally occurring pro-resolving mediators and their receptors offers new potential targets for drug design and the opportunity to investigate the underpinning molecular mechanisms of inflammation resolution.

Could life-style factors play a role in the epidemic of non-communicable and age-related diseases and the associated pro-inflammatory phenotype? Evidence exists that suggests that the Mediterranean diet which includes olive oil and some omega-3 lipids, can ameliorate RA (38), may give some protection from atrial fibrillation and myocardial infarction (MI) (39), and improves diabetic control (40). Research has also demonstrated a protective role of the Mediterranean diet in gene/Mediterranean diet interactions for the risk TT allele of the TCF7L2-rs7903146 gene in stroke risk and mortality (41, 42). Improving knowledge about how inflammation shuts down in a timely way is crucial to the understanding of how chronic inflammation contributes to aging and age-related diseases. Further studies are likely to be needed to advise if dietary modifications with omega-3 lipids or whether synthetic resolving mimetics are part of the answer.

## Triggers of the Inflammation Pathway

Several common molecular pathways have been identified that seem to be associated with both aging and low-grade inflammation. These pathways trigger the inflammasome, stimulating NF-κB, and the IL-1β-mediated inflammatory cascade.

### Age-Related Redox Imbalance

A redox imbalance has long been associated with aging and led to the development of the redox stress hypothesis of aging (43). Redox stress is caused by an imbalance between unregulated and overproduced reactive oxygen species (ROS) that are produced secondary to mitochondrial energy production, active immunological phagocytic processes, and the prostaglandin pathway through COX enzyme production. While ROS are important molecules regulating numerous physiological and pathological processes in the cell, there is now clear evidence that overproduction of ROS is involved in the development of a number of diseases, such as Alzheimer’s disease, rheumatoid, and cardiovascular diseases. Increasing evidence supports the notion that low concentrations of ROS or “primary ROS” are involved in well controlled processes (44), where their effect on reactive target molecules can be reversible, suggesting that “primary” ROS acts as an important intracellular signaling molecule (45). In contrast, the very active OH ROS is less effectively controlled and forms the main damaging type of ROS that is able to react with many macromolecules, such as lipids, proteins, and nucleic acids. This results in DNA oxidation and cell membrane damage, which contributes to the burden of damaged molecules related to aging and age-related diseases.

#### Mitochondrial ROS

Mitochondria are highly efficient producers of energy, but in doing so they produce ROS. It is estimated that about 90% of intracellular ROS is generated in the mitochondria through the mitochondrial transport chain. The chain of electron flow is considered to leak prematurely between complexes 1, 11, and 111 leading to the formation of damaging oxidants like ${\text{O}}_{2}^{-}$. This ROS has been considered to cause damaging mutations in the mitochondrial genes with increasing age (43). With increasing age, mitochondrial function becomes sluggish and this compromises energy production, which in turn further contributes to mitochondrial dysfunction (46). A vicious cycle develops with age-reduced physical activity producing muscles that become weaker, are infiltrated with fat cells, and show less efficient mitochondria energy production (47). Ischemia and apoptosis can trigger ${\text{O}}_{2}^{-}$, and mitochondria themselves can be damaged by ROS production. Mitophagy, the removal of damaged mitochondria is also reduced as age increases (48). A reduced age-related capacity of the body’s anti-oxidative defense systems to mop up free radicals also plays an important role in maintaining the inflammatory background of chronic inflammation (49).

#### The Nicotamide Adenine Dinucleotide Phosphate (NADPH) Pathway of ROS

One of the other main producers of ROS is the specialized enzyme group of the NADPH oxidases of the NOX family—(NOX1, NOX2, NOX3 NOX4, NOX5, DUOX1, and DUOX2). The NOX family or NADPH oxidases’ generate ${\text{O}}_{2}^{-}$ or H_2_O_2_ radicals by transferring electrons from cytoplasmic NADPH or the “NOX” catalytic subunit to molecular oxygen (50). The ROS produced by these enzymes has an essential function in neutrophils and macrophages as a mechanism for effective bacterial killing and host defense (51, 52). When the phagocytes sense an endogenous or exogenous danger signal, the NADPH-oxidase unit translocates to fuse with the plasma membrane to form the phagosome. This generates large amounts of highly reactive ROS called the phagocytic burst that is very effective in killing microbes, though phagosomal pH and ion concentration are also likely to be contributors.

Although NOX family of isoenzymes was initially associated with the ROS produced in phagocytes, other members of the NOX family are now known to be involved in a wide range of regulatory functions in many tissues and seem likely to play a role in aging and age-related diseases. Studies in the human vascular system suggest that NOX1, NOX2, and NOX5 promote endothelial dysfunction, inflammation, and apoptosis in the vessel walls, whereas NOX4 by contrast is vasoprotective, by increasing nitric oxide bioavailability (53). NOX enzymes, therefore, appear to play a role in vascular pathology as well as in the maintenance of normal physiological vascular function. Activation of NOX2 and NOX4 occurs in humans with atrial fibrillation and inhibition of NOX by angiotension converting enzyme inhibitor drugs or statins has proved helpful in preventing post-operative atrial fibrillation (54).

#### COX Pathways of ROS

The biolipids are highly reactive substances that contribute to both inflammation and healing and their pathways produce and use ROS signaling. The reaction that converts AA through COX2 into prostaglandin H2 (PGH_2_) by a two-stage free radical mechanism (55) involves superoxide and can contribute to cellular oxidative stress as well as signaling. Other enzymes that generate ROS during AA metabolism include the arachidonic 12-lipoxygenase (LOX-12 or ALOX12) and arachidonic 5-lipoxygenase (LOX5 or ALOX5), both of which also activate and induce NADPH-oxidases (56).

While mitochondrial ROS are traditionally seen as the main source of intracellular ROS and, therefore, major mediators of ROS-induced damage, the relative contribution of mitochondrial and non-mitochondrial sources of ROS to induction of cellular senescence remain unclear. Both mitochondrial ROS and NADPH-produced ROS appear to be able to cross signal between each other and mitochondria have significant antioxidant capacity, which may act as a cellular redox buffer for NADPH-produced ROS, suggesting there is tight control and integration of ROS signaling within the cell.

The cellular systems that protect against ROS, include the anti-oxidative defense enzymes, superoxidase dismutase, glutathione peroxidase, and catalase (57), oxidant scavengers (vitamin E, vitamin C, carotenoids, uric acid, and polyphenols), and mechanisms to repair oxidant damage to lipids, proteins, or DNA. Despite these protective mechanisms, uncontrolled ROS can overwhelm the antioxidant capacity of the cell causing mitochondrial dysfunction (49). Increased ROS production from the various cellular sources stimulates intracellular danger-sensing multi-protein platforms called inflammasomes (58–60). Through the inflammasome, the ROS activates NF-κB which sets in motion the transcription of a cascade of pro-inflammatory cytokines—tumor necrosis factor-alpha (TNF-α), IL-1β, IL-2, and IL-6, chemokines—IL-8 and RANTES, and adhesion molecules, such as ICAM-1, VCAM, and E-selectin, that are central mediators in the inflammatory response.

### Autophagy Slowing and Aging

Approximately a third of all newly synthesized proteins are formed in the endoplasmic reticulum (ER), where they are folded, modified, sorted, and transported to sites where they perform specialized roles. Stressors, such as low glucose as in fasting, alterations in calcium levels, low oxygen states, viruses, cytokines, and nutrient excess or deficiency can trigger the autophagy pathway with the aim of returning normal homeostasis to the cell.

Autophagy is a cellular process whereby cellular waste, such as modified proteins, protein aggregates, and damaged organelles are removed from the cell. It is a tightly controlled process that plays a role in growth and development and maintains a balance between the synthesis, degradation, and subsequent recycling of cellular products. Autophagy can be considered a protein and organelle quality control mechanism that maintains normal cellular homeostasis.

**Figure 3:**
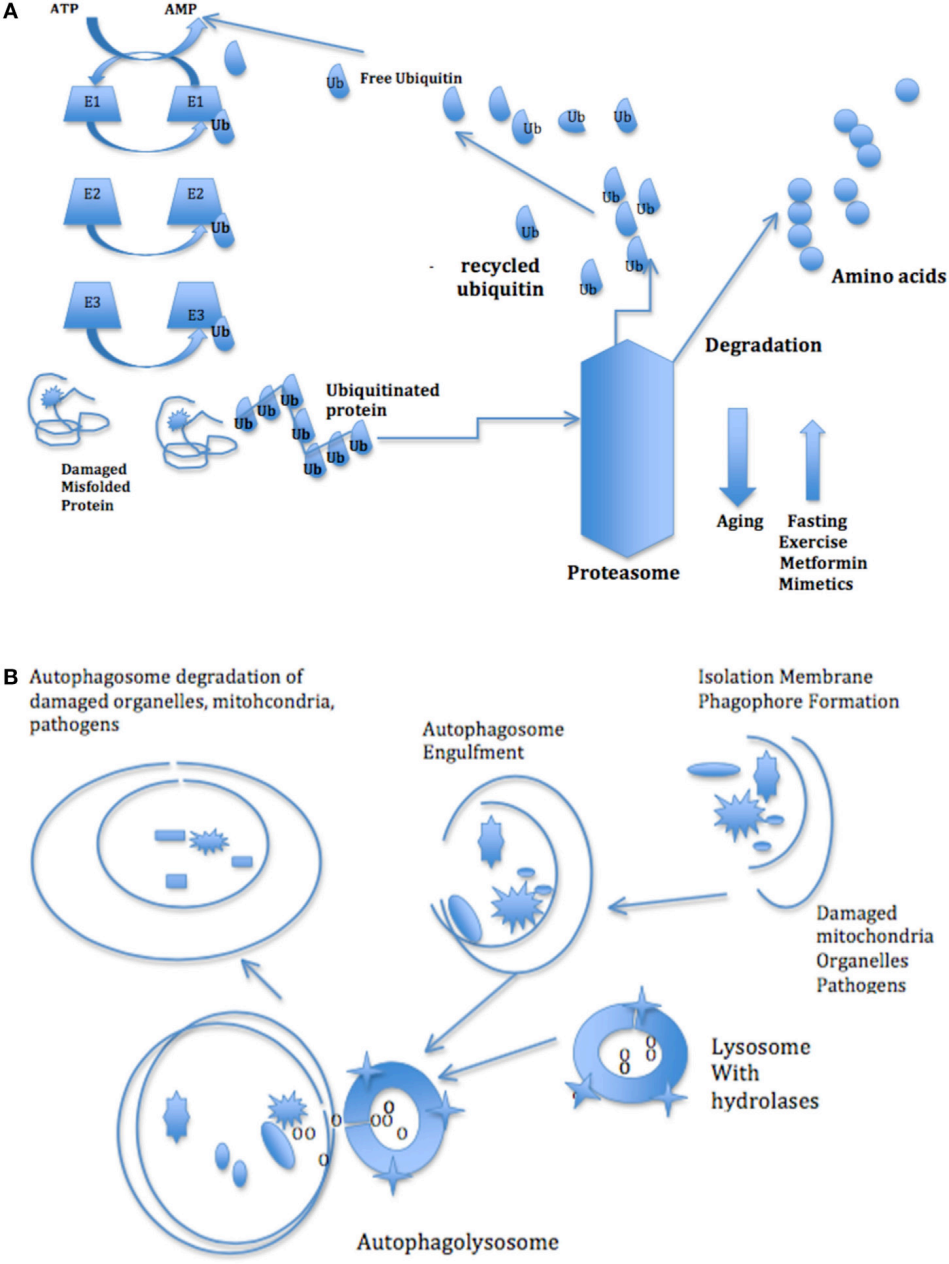
**(A)** The ubiquitin–proteasome pathway of protein degradation. Three different sets of enzymes—E1, E2, and E3, identify and categorize proteins in order to link ubiquitin or ubiquitin complexes to the damaged proteins. The ubiquitin-protein complexes pass through the proteasome where they are degraded and discharged as free amino acids into the cytoplasm. **(B)** The autophagy pathway of degradation of damaged organelles and pathogens. The autophagy system degrades larger aggregated proteins and cellular organelles, such as mitochondria, peroxisomes, and infectious organisms. The process involves membrane formation, fusion, and degradation. A small separate membrane called a phagophore forms and then forms the autophagosome that fuses with the lysosome. The autophagosome contents are degraded by lysosomal hydrolases.

Two major pathways degrade cellular proteins. The ubiquitin-proteasome system (UPS) degrades 80–90% of denatured and damaged proteins. In the ATP-dependent UPS, damaged or misfolded proteins are tagged with a small protein called ubiquitin. Three different sets of enzymes—E1, E2, and E3, identify and categorize proteins in order to link ubiquitin or ubiquitin complexes to the damaged proteins. The ubiquitin-protein complexes pass through the proteasome, where they are degraded and discharged as free amino acids into the cytoplasm (Figure 3A).

The other main pathway is the autophagy system that degrades cystolic components, including larger aggregated proteins and cellular organelles, such as mitochondria, peroxisomes, and infectious organisms (61). This process involves membrane formation, fusion, and degradation (Figure 3B). When autophagy is induced, a small separate membrane structure called a phagophore arises in the cytoplasm, which gradually expands to form the autophagosome. The outer membrane of the autophagosome fuses with the lysosome and the autophagosome contents are degraded by lysosomal hydrolases (62). Like the proteasome, the macroautophagy system is stimulated by intracellular and extracellular stress-related signals, including oxidative stress. Both proteasome and autophagy produce small polypeptides that help maintain a pool of amino acids and control energy balance in starvation, since recycling amino acids is more energy efficient than *de novo* amino acid synthesis.

In aging and age-related disease there are gradual reductions of cellular repair mechanisms that lead to the accumulation of damaged molecules, proteins, DNA, and lipids leading to loss of efficient cellular function. The cell’s capacity for autophagic degradation also declines with age and this in itself may contribute to the aging process (63). While both major systems for intracellular protein degradation are slowed up with increasing age, a physical reduction of autophagy-related proteins also contributes to the accumulation of misfolded proteins and damaged macromolecules in the cell. Diseases associated with increased oxidative stress, such as cardiovascular and Crohn’s disease and obesity also slow up cellular clearing and reduce autophagy, further contributing to disease (64–66).

The lysosome–autophagy system carries out a wide range of non-specific intracellular degradation and cleaning processes, which include managing pathogens, damaged intra-cellular macromolecules, and surface receptors (67–69). Lysosomal dysfunction is associated with age-related pathology that reduce lifespan, such as Parkinson’s and Alzheimer’s diseases (70, 71). Senescent cells accumulate abnormal protein aggregates in the cytoplasm, and contribute to neurodegenerative disease (72).

The dysregulation in autophagy has important effects in the innate immune response, in aging and age-related diseases by influencing inflammasome activity, cytokine secretion, antigen presentation, and lymphocyte function (73, 74). Under normal circumstances the nod-like receptor 3 (NLRP3) inflammasome fine-tunes the progression of the innate immune response that it has initiated, by upregulating autophagy activity so that the removal of immune mediators is expedited (74). In aging and age-related diseases, the autophagy response becomes blunted, the immune mediators remain active and prolong the inflammatory response (75).

The UPS and autophagy act synergistically and cooperatively to maintain cellular homeostasis (76). Effective autophagic uptake of dysfunctional mitochondria and efficient lysosomal degradation of damaged aggregated proteins and macromolecules are crucial elements in maintaining tissue homeostasis and good health (77). The decline in the autophagy capacity, that impairs cellular housekeeping in aging, seems to be an attractive molecular pathway to target to improve the quality of aging.

Two groups of drugs, the mammalian target of rapamycin (mTOR) inhibitors and AMP-activated protein kinase (AMPK) activators are promising pharmacological agents which stimulate autophagic degradation (78–80). Other drugs, such as the diabetic drug metformin and the oncology agent 5-aminimidazole-4-carboxamide ribonucleoside are pharmacological activators of AMPK, which are soon planned for clinical studies in relation to aging (81–83). A number of substances, such as curcumin, berberine, and quercetin, regularly available in normal diets, appear able to mimic the action of AMPK and upregulate autophagy. The action of AMPK has important anti-inflammatory and immunosuppressive effects (83). By upregulating autophagic activity, AMPK promotes effective clearing of DAMPs and by preventing the activation of the inflammasome, it reduces the triggering of the inflammatory cascade. Further evidence of the anti-inflammatory role comes from research with the AMPK agonist A-769662 that mimics AMPK activity (84). This AMPK mimetic has been shown to suppress inflammatory arthritis in mice and reduce IL-6 expression in serum and arthritic joints, suggesting that targeted AMPK activation could be an effective therapeutic strategy for IL-6-dependent inflammatory arthritis (85).

Non-pharmacological life-style changes also upregulate autophagy. One of the best researched is the effect of exercise which improves mitochondrial mitogenesis and stimulates mitogeny, so improving the quality of muscle function and exercise performance, with improvement in the quality of aging (86, 87). Furthermore in animal model studies, both modulated caloric restriction and exercise increase autophagy, downregulate endotoxin-induced IL-1β production, improve the aging-related pro-inflammatory profile, and reduce disease symptoms (78, 88).

Further understanding of molecular pathways of the signaling networks underpinning autophagy should help to identify other novel drug targets. Important research areas include those that could improve the sensitivity of degradation inhibitors useful to improve anticancer treatment, or new drugs to upregulate autophagy to maintain good cellular housekeeping, with the potential for improving the quality of aging and the management of age-related degenerative diseases.

### Senescent Cells

Senescent cells increase with age and are considered important contributors to the pro-inflammatory phenotype (89). The two major hallmarks of cellular senescence are an irreversible arrest of cell proliferation and production of the pro-inflammatory secretome, called the SASP. When replicative senescence was first identified in serial cell passage studies (90), telomere attrition was considered to cause the cellular growth arrest that acted as a mechanism to stop damaged or transformed cells from proliferation and transiting to tumor initiation. Today senescence is considered to have much broader role as both a contributor to damage protection and in the control of cellular growth, or as both a “friend and foe” depending on the cellular context. Senescence together with apoptosis is recognized to play an important physiological role in normal embryonic development, in ongoing tissue homeostasis throughout life (91, 92), but is increasingly considered to have a role in causing or exacerbating aging and age-related diseases (91, 93–95).

Senescence is a stress response triggered not only by telomere attrition as originally described (90, 96), but also by stress insults, such as genomic instability, DNA damage, protein misfolding and/or aggregation, and ROS. There is also an association between senescent cells and the dysregulated mitochondrial network and associated metabolic dysfunction that is seen with increasing age (97). Through the SASP, the senescent cell has an important influence on the extrinsic microenvironment, which suggests a link between senescence and alterations in intracellular and intercellular communications (93).

Cells that express senescence markers accumulate with age in some tissues in studies in mice and man (98–100). Senescent cells are found in association with age-related diseases, such as atherosclerosis, RA, neurodegenerative diseases, and cancer (101–104). In RA patients T-cells are described as showing a pre-aged phenotype with apparent loss of CD28 expression that reduces T-cell activation and this in association with reduced RA-related NK surveillance, could allow senescent cells and the associated SASP to persist. In cancer, SASP factors promote angiogenesis, cell proliferation, and cancer invasiveness. Cells attracted by SASP influence the local microenvironment with the potential to promote tumor invasion and cancer progression (105). Senescent cells have been seen in atherosclerotic plaques (101). Recent data from several laboratories has suggested that both aging and age-related neurodegenerative diseases show an increase in SASP-expressing senescent cells of non-neuronal origin in the brain, which correlated with changes in neurodegeneration (103).

The SASP consists of a complex combination of growth factors, proteases, chemokines, matrix metalloproteinases, and is particularly enriched in pro-inflammatory cytokines, especially IL-6 (106–108). The SASP-secreting cells respond by switching on a self-perpetuating intracellular pro-inflammatory signaling loop, centered around the NF-κB, TGF-β, IL-1α, IL-6 pathway (109–111), with suggested mechanisms related to higher basal phosphorylation and altered threshold signaling (112) or alternative splicing (113). Senescent cells influence other cells by paracrine and bye-stander effects (114). There appears to be multi-level control of senescence and the SASP secretome, which includes the tumor suppressor pathways involved in the cell cycle arrest and the NF-κB and persistent damage response (DAMP) pathway, involved in triggering transcription of the SASP-related factors (115). Several pathways of investigation suggest that senescent primary human CD8+ T cells use anaerobic glycolysis to generate energy for effector functions and that p38 mitogen-activated protein kinase (p38 MAPK) blockade may reverse senescence *via* the mTOR-independent pathway (116). Low doses of glucocorticoid suppress elements of the SASP in patients with RA and improve clinical symptoms (117). Senescent cells effectively recruit the immune system to organize their removal, but with increasing age, removal becomes sluggish or otherwise impaired (118, 119).

It can be argued that the increase in senescent cells with aging reflects either an increase in their rate of generation or a decrease in their rate of clearance because the immune response is attenuated or weakened with aging and less capable of clearing senescent cells (120–122). Senescent cells express ligands for cytotoxic immune cells, such as natural killer (NK) cells, and have been shown to be able to be specifically eliminated by the immune system (123, 124). Through a proteomics analysis of senescent cell chromatin, the NF-κB pathway appeared to act as a master regulator of the SASP, with NF-κB suppression causing escape from immune recognition by NK cells (125). Other studies show that processes which eliminate senescent cells with p16(Ink4a)-positive markers, delay age-related pathologies in the mouse model of aging though side-effects can be problematical (126, 127). Therapies that specifically recognize and trigger the elimination of senescent cells would seem important to enhance the immune system in older people. New methods are in the process of being developed to enhance the immune clearance and autophagy of the increased senescent cell burden in aging and age-related disease (128).

### Inflammasome NLRP3

**Figure 4:**
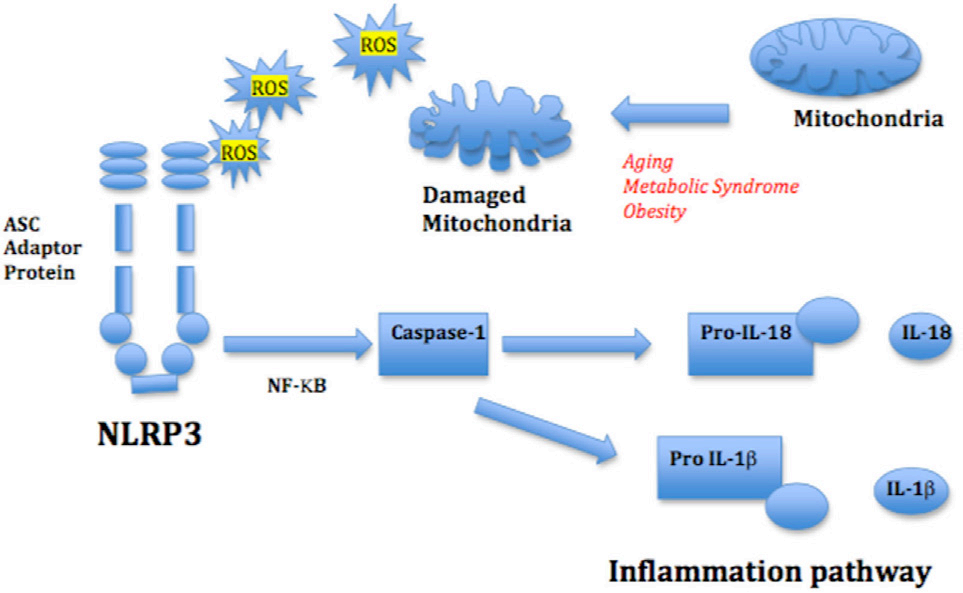
Mitochondrial reactive oxygen species (ROS) and nod-like receptor 3 (NLRP3) activation of inflammation pathway. Mitochondrial ROS from damaged mitochondria triggers the inflammasome NLRP3, stimulating NF-κB and the IL-1β and IL-18-mediated inflammatory cascade. The adapter protein ASC mediates innate signaling by bridging the interaction between the damage recognition receptor and the NF-κB caspase-1 inflammasome complex.

The inflammasomes, intra-cellular multiprotein sensors that recognize danger signals, are likely key players in initiating and maintaining the pro-inflammatory phenotype found associated with aging. The NLRP3 is a major inflammasome sensor for intracellular stress molecules called DAMPs, which together with damaged aggregated proteins that are released from destabilized lysosomes and damaged mitochondria contribute to the cellular stress (ROS) and trigger NLRP3 activation (129). Once activated, the NLRP3 inflammasome initiates the inflammatory response cascade by stimulating caspase-1 (casp-1) that acts to induce the active precursors of pro-inflammatory cytokines, such as IL-1β, IL-1α, and IL-18, and on-going interaction with NF-κB (130, 131) (Figure 4). Although the baseline activity of NLRP3 is low, the initiation process of the inflammatory cascade requires a complex oligomerization-priming phase that includes association with NF-κB and so contributes several layers of regulatory control.

Nod-like receptor 3 has been shown to be able to activate NF-κB and induce cytokines in response to sterile signals, such as monosodium urate crystals and aluminum adjuvant, suggesting that NLRP3 could initiate NF-κB activation to both pathogen-induced and sterile inflammation (132). Conversely NF-κB, which primes the NLPR3 inflammasome for activation also prevents excessive inflammation and restrains NLRP3 activation by enhancing the NF-κB-p62 mitophagy pathway. By self-limiting the host response, the NF-κB-p62 mitophagy pathway maintains homeostasis which under normal conditions leads to tissue repair (75). It is, however, unclear if this layer of control of NF-κB function remains as tightly controlled in aging and age-related disease.

The NLRP3 inflammasome is a key component of the innate inflammatory response to pathogenic infection and tissue damage. It responds to a wide range of cellular stress and is considered to contribute to the aging process and to age-related diseases (133). Zhou and colleagues identified that mitochondrial ROS was involved in the activation of NLRP3 (58). This study emphasized the important role of mitochondria in maintaining a correct balance between cellular energy production and ROS production and that effective clearance of damaged mitochondria through autophagy was an important regulatory activity. Damaged mitochondria increase with aging and age-related diseases (134). Mitochondrial dysfunction drives mitochondrial mutagenesis, affecting respiratory chain genes, and compromising the efficiency of oxidative phosphorylation, which may lead to further mt-DNA mutations and more cell damage. The subsequent mitochondrial impairment leads to more ROS that further reduce ATP generation and increases the chance of cell death. Mitochondria have been identified as a key source of DAMPs, the so-called mito-DAMPs, which have been considered to play a role in DAMPS-modulated inflammation in diseases, such as RA, cancer, and heart disease (135–138) as well as in the aging process (139). Degraded mt-DNA has also been reported in neuroinflammation (140). Dysfunctional mitochondria seem to be able to initiate an auto-feedback loop to increase autophagy, so that damaged mitochondria or misfolded proteins are degraded which reduces inflammasome activation and risk of further tissue injury, though this system is less efficient in aging (141).

Lyosomal destabilization is also associated with NLRP3 activation and can be induced by a number of molecules, including cholesterol crystals in macrophages linking atherosclerosis progression with inflammation (142). There is deposition of other harmful intra- and extracellular material in several age-related diseases. The aggregates compromise cellular homeostasis and can provoke the activation of the NLRP3 inflammasome. Research has shown that amyloid fibrils and Alzheimer’s amyloid-β can trigger NLRP3 inflammasomes and in that way stimulate inflammation and enhance pathogenesis and association between type 2 diabetes and Alzheimer’s disease, respectively (143). Palmitate, a saturated fatty acid has been shown to activate NLPR3, whereas oleic acid did not initiate the same inflammatory response (144). The inflammasome has been implicated in the development of the metabolic syndrome through impairment of adipose tissue sensitivity. Evidence showed that obesity triggered NLRP3 activation, and that the secreted IL-1β impaired insulin signaling which promoted insulin resistance in mice (145). Other research has shown that obesity was associated with the activation of the NLRP3 in adipose tissues (146, 147).

A number of intracellular processes seem likely to work together to stimulate and augment the inflammasome pathway and contribute to pro-inflammatory cytokine upregulation associated with increased age and age-related diseases. Both the redox-sensitive inflammatory pathway and the senescent cell-related SASP activate the inflammasome through the NF-κB and IL-α cascade, causing persistence of the inflammatory response that delays resolution and healing (125, 138). Similarly, reduced autophagy processes allow the accumulation of damaged intracellular proteins and senescent cells that further perpetuate and amplify the pro-inflammatory milieu that is found with increased age and is associated with age-related diseases.

## Pro-Inflammatory and Anti-Inflammatory Cytokine Dysregulation

### Pro-Inflammatory Cytokines in Aging and Age-Related Disease

**Figure 5:**
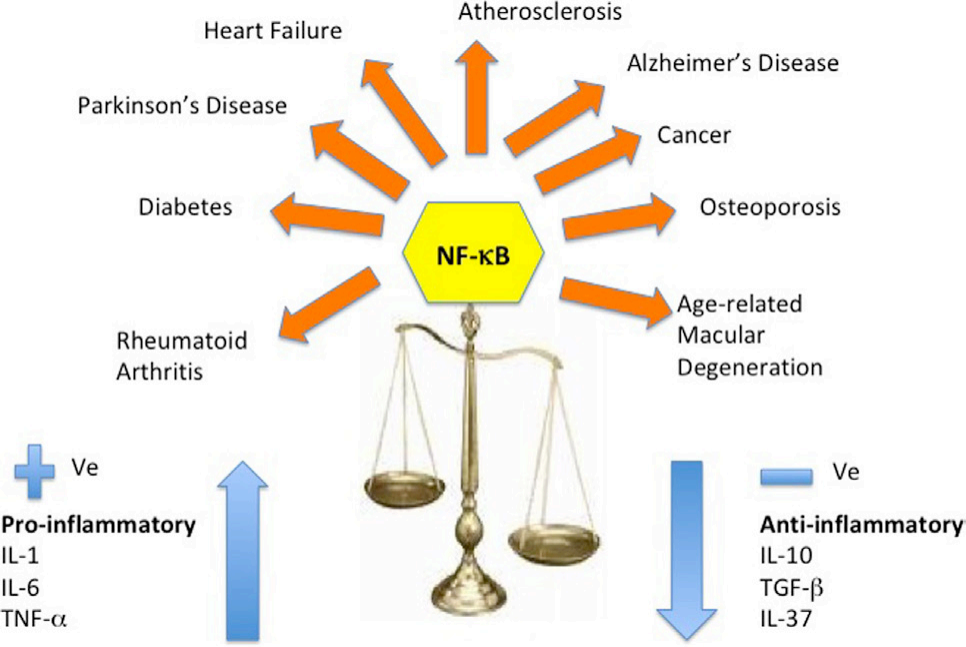
Cytokine dysregulation and NF-κB inflammation pathway. This reshaping of cytokine expression pattern with a progressive tendency toward a pro-inflammatory phenotype has been called “inflamm-aging” and is found associated with age-related diseases. Several molecular pathways have been identified that trigger the inflammasome and stimulate the NF-κB and the IL-1β-mediated inflammatory cascade of cytokines.

Various biomarkers and biochemical indices are used in medicine and age-related diseases as a way of improving diagnosis, beyond the well-recognized clinical signs. Modest increases in concentration of C-reactive protein, a circulating marker of inflammation, have been widely reported to be associated with a large number of age-related conditions and lifestyles felt to be associated with poor health; these conditions represent or reflect minor metabolic stresses. Alongside C-reactive proteins, cytokines have come under investigation as the molecular processes and pathways underpinning inflammation have become better identified. A common finding in aging and age-related diseases is “inflamm-aging,” a dysregulation of the cytokine network and its homeostasis. Downstream from NF-κB signaling, the pro-inflammatory cytokines play a central role in the remodeling of the immune system with age (Figure 5).

The major pro-inflammatory cytokines, such as IL-6, TNF-α, and IL-1α contribute significantly to the phenomenon of inflamm-aging in healthy elderly individuals (8), while also playing a major role in many age-related diseases (11, 27, 148–151). The key to healthy aging must lie in the ability to maintain a balanced response to these immune messengers and a prompt and integrated return to inflammation resolution and immune homeostasis (17). A summary of the changes that have been described in pro-inflammatory and anti-inflammatory cytokines in aging and some age-related diseases are outlined in this section.

#### Interleukin-1 (IL-1) Family

IL-1α and IL-1β, known as IL-1, and IL-18 are important cytokine initiators of the stress-induced inflammatory cascade (152). IL-1β and IL-18 are cleaved to active forms by Casp-1, whereas IL-1α is activated by calpain protease. All bind to and activate the IL-1R that is downregulated by the receptor anatagonist IL-1Rα, which blocks IL-1-mediated signal transduction.

Studies in elderly people, including centenarians have reported an age-related rise in the IL-1R antagonist, (IL-1Rα), whereas IL-1β showed no detectable age-related trend. The age-related rise is associated with increased co-morbidity, age-related disease, and mortality (153–156).

Certain IL-1 haplotype-carriers produce increased IL-1β, and IL-1 gene variations associate with earlier onset or more severe progression of cardiovascular and Alzheimer’s disease, but not with osteoporosis (157–161). In centenarians, no single IL-1 gene polymorphism showed a survival advantage, but in Swedish elderly males an IL-1 gene polymorphism shortened life expectancy (153, 162, 163). IL-1 gene variants appear to increase the risk of age-related diseases and recombinant drugs, such as IL-1Rα-blockers may have a role in the clinical control of inflammation (164).

#### Interleukin-18 (IL-18)

Interleukin-18, a linked IL-1 pro-inflammatory cytokine, signals in a complex with IL-18 receptors α (Rα) and β (Rβ) chains and induces IFN-γ that is essential for defense against infections (165). IL-18’s multiple pro-inflammatory effects are modulated through IL-18 binding protein (166).

Higher levels of IL-18 have been found in centenarians, associated with heart failure, ischemic heart disease, and type 1 diabetes in patients, and in the Alzheimer’s disease brain (167–172). IL-18 levels associate with physical functioning and with a frailty index in the English longitudinal study of aging, where carriers of IL-18 gene polymorphism that reduced IL-18 levels, showed improved walking speed (173–175). Evidence consistently shows that IL-1 and IL-18 are mediators of inflammation and associated with the aging process (168). Drugs blocking binding between IL-18 and the receptors are currently in development and may provide benefit in the treatment in diabetes, macular degeneration, and autoimmune disease (176).

#### Interleukin-6 (IL-6)

Interleukin-6 has been long recognized as important in aging and age-related disease and has been called the “gerontologist’s cytokine” (177, 178). IL-6 plays a key role in the acute phase response, in the transition from innate to acquired immunity, in metabolic control, and in the pathogenesis many chronic diseases (11, 148–151, 179). It has both pro- and anti-inflammatory activities, and modulates the acute inflammatory response by producing IL-1 Rα and soluble tumor necrosis factor receptor p55 (sTNF-R55), which suppresses TNF-α and IL-1.

Interleukin-6 is normally present in low levels in the blood, but is increased in aging and in subjects with markers of frailty and chronic disease, where it tracks with mortality (180–183). IL-6 is a risk factor associated with cardiovascular disease and is associated with sarcopenia and muscle loss (184, 185).

The G allele of IL-6-174C/G polymorphism shows higher IL-6 levels and associates with cognitive decline and mortality in age-related vascular disease, whereas CC allele carriers show decreased Alzheimer’s risk (186–191). In a meta-analysis of longevity in a large cohort of European nonagenarians and centenarians there was longevity benefit for carriers of the lower cytokine producing IL-6 allele, with similar supporting findings for this IL-6 allele in a case control study (192, 193). IL-6 or IL-6 receptor blockers are already used successfully in the treatment of RA, and are proof of concept that damping down IL-6, a product of the NF-κB pro-inflammatory cascade, can improve clinical symptoms. Studies are either in progress or planned to assess the outcome of blocking IL-6-related inflammation in other age-related diseases with the potential for contributing to more successful aging (194, 195).

#### Tumor Necrosis Factor Alpha

Another major player in the immune response is the pro-inflammatory cytokine TNF-α, which increases with age and is associated with age-related disease (196). It is a pro-inflammatory mediator that can be beneficial when it acts locally in the tissues, but can be highly harmful when released systemically.

Tumor necrosis factor-α has been reported to be increased in intracellular aging studies in elderly people, in centenarians and octogenarians with atherosclerosis, and associated with mortality (197–202). In post-MI patients, a rise in TNF-α increased risk of recurrent cardiac events and in renal patients TNF-α receptors predicted cardiovascular disease (203–205). In genetic studies, the A allele of TNF-α 308 G/A gene associated with risk for MI, whereas TNF-α polymorphisms and TNF-α itself, have been variably associated with increased Alzheimer’s disease risk (206–210). TNF-α mediates metabolic changes and increased TNF-α was found in type 2 diabetes mellitus and was associated with lower muscle mass and strength in older groups (211).

In studies in nonagenarian/centenarian groups from three European countries, there was no attrition of the TNF-α-308 A/G polymorphism in centenarians (162, 212, 213). With increasing evidence of an association between increases in TNF-α and age-related diseases, research re-purposing anti-inflammatory drugs are under development. Research has demonstrated that TNF-α inhibitors may have possible prophylactic or ameliorating roles in cardiovascular and Alzheimer’s disease in animal models (214, 215).

#### Other Pro-Inflammatory Cytokines

Other pro-inflammatory cytokines are increasingly being recognized as dysregulated in association with aging and age-related disease.

##### Interleukin-2

Interleukin-2 plays a pivotal role in the immune response. It is a growth factor that promotes NK cell activity and the differentiation of naïve T cells into Th1 and Th2 cells (216). Conversely, IL-2, acting *via* STAT5 pathway negatively regulates interleukin 17 (IL-17) production (217). Most studies show that lymphocytes in elderly people produce significantly less IL-2, compared to young people (218–220). Intracellular cytokine studies have shown variable results for IL-2, whereas mitogen-induced stimulation of mononuclear cells from elderly subjects showed significant decreases in IL-2 and IFNγ production (197, 221).

##### The IL-7/IL-7R

The IL-7/IL-7R network is essential at various stages in T-cell development and survival (222). It has an important role in the maintenance of a vigorous health span and higher IL7R gene expression is associated with long life (223–225). Serum IL-7 is increased in some age-related diseases, including osteoarthritis and genetic variation in the IL7RA/IL7 pathway increased susceptibility to multiple sclerosis (226, 227). Research has suggested that silencing of the IL-7R gene may be an important mechanism underpinning an aging-related loss of binding to NK-κB (228), linking IL-7R gene to the NF-κB pathway and inflammation control.

##### Interleukin-12

Interleukin-12, a pro-inflammatory member of the IL-6 family has an active role in the development of cardiovascular diseases, such as atherosclerosis, MI, and stroke (229). Patients with cardiovascular disease show increased levels of IL-12, 23, and 27 with higher IL-12 predicting poorer long-term outcome after acute MI (230). Other research shows variable results for IL-12 and its receptor antagonist, with increased IL-12 (total) and IL-12p40 in apparently healthy nonagenarians, lower IL-12p70 and IL-23 production in association with frailty and IL-12/23p40 ameliorating Alzheimer’s disease in animal models (231–233).

##### Interleukin 17

Interleukin 17 is a key pro-inflammatory cytokine that belongs to a family of six cytokine members (A–F). IL-17A (referred to as IL-17) plays a central role in host defense against invading pathogens and is produced by a subset of CD4+ cells (234, 235). Elderly people (age ≥65) have shown a decreased frequency of IL-17-producing cells in memory subset of CD4+ T cells compared to healthy younger people (236). IL-17 enhances production of IL-6, TNF-α, the acute phase reactants, C-reactive protein, and serum amyloid A and activates the induction of IL-6, IL-8, and G-CSF in non-immune cells, such as fibroblasts and epithelial cells, in part through activation of the NF-κB transcription factor (237). IL-17 promotes inflammation and is overexpressed in many autoimmune diseases, such as RA, systemic lupus erythematosus, inflammatory bowel disease, and psoriasis and its effects are stabilized by IL-23 (238–241). An IL-17 expressing CD8+ T subset of cells has also been reported to be involved in psoriatic arthritis and some other autoimmune diseases (242, 243).

##### Interleukin-8

Interleukin-8 (or CXCL8) is a chemokine secreted by monocyte/macrophages whose key role in the inflammation process is the recruitment and activation of neutrophils. IL-8 has been implicated in a number of inflammatory conditions, such as cystic fibrosis, asthma, chronic pulmonary disease, inflammatory bowel disease, and some autoimmune diseases, including RA and psoriasis.

Increased levels of IL-8 have been detected after LPS-stimulation of leukocytes from elderly individuals (244). In one small study of centenarians, IL-8 was proposed as a possible longevity factor (245). A single study of IL-8 polymorphisms found no significant difference in IL-8 -251 A/T polymorphisms in nonagenarians compared to young controls (212). IL-8 signaling occurs *via* the MAPK and PI3K pathways, by binding to the IL-8 receptors-CXCR1/2. Several agents that block IL-8-CXCR1/2 signaling have been developed in an attempt to target inflammatory pathways in cancer, asthma, chronic obstructive pulmonary disease, psoriasis, and RA (246).

### Anti-Inflammatory Cytokines in Aging and Age-Related Disease

The anti-inflammatory cytokines play a key role in balancing the immune response, and in preventing the tipping of the steady state of immune homeostasis across into inflamm-aging and a disease-inducing state. Anti-inflammatory cytokines are an important arm of inflammation resolution. They block or modulate the synthesis of IL-1α, TNF, and other major pro-inflammatory cytokines and damp down the inflammatory response, so that inflammation resolution can begin. Specific cytokine receptors for IL-1, TNF-α, and IL-18, together with soluble receptor antagonists, chemokines, microRNA, siRNAs, also function as inhibitors for pro-inflammatory cytokines. The anti-inflammatory cytokines and families of soluble receptor antagonists work within a complex network of control of immune regulation. They are critical for balancing the inflammatory outcome and together with pro-resolving lipoxins are critical to resolving inflammation in an integrated and organized manner.

As age increases and in age-related diseases, a chronic inflammatory state predominates, which is not properly contained or resolved and the anti-inflammatory side of the immune system seems to be similarly dysregulated, and unable to damp down the inflammatory episode in a timely effective manner. The following cytokines are the major players in the anti-inflammatory pathway of the control of inflammation and changes in their production and expression have been quite widely reported in aging and age-related disease. Where increases in anti-inflammatory cytokines have been reported, one interpretation would be that increases might reflect the immune system’s attempt to suppress the persistent pro-inflammatory response and support a return to immune homeostasis.

#### IL-10 Family

Interleukin 10 is one of the key anti-inflammatory cytokines, which suppresses the actions of IL-6, TNF-α, and IL-8 (247, 248). Higher IL-10 serum levels and production by both lymphocytes and monocytes have been reported in elderly people (155, 244, 249). Conversely an age and gender-related decline in cellular stimulation studies has been reported (250).

In age-related disease, IL-10 has been reported to be associated with vascular protection in atherosclerosis and improved endothelial dysfunction (251–253). However, at variance, the authors from the ERA (254) and PROSPER (255) studies, concluded that elevated IL-10 increased cardiovascular risk among elderly groups, and suggested that IL-10 blockers merited investigation. In male Sicilian centenarians, male carriers of the high producing GG 1,082 allele of the IL-10 promoter polymorphism showed a survival advantage, suggesting that IL-10 anti-inflammatory activities might be a marker for male longevity (213). This result was not replicated in Sardinian, Irish, or Finnish nonagenarian/centenarians (162, 212, 256). It has been argued that an enhanced anti-inflammatory phenotype could be beneficial and contribute to longevity by controlling the pro-inflammatory milieu that predominates in later life and contributes to increased morbidity and mortality (9, 11, 257).

#### TGF-β

TGF-β, another important anti-inflammatory cytokine limits both the acute phase response, and is involved in tissue repair post-damage or infection (258). Several authors have reported that TGF-β was increased in octogenarians and centenarians (148, 259). It is also involved in aging-related disease, such as in obesity, in vascular wall integrity, in muscle loss and sarcopenia, in osteoarthritis, and with frailty in the Newcastle longitudinal study (260–264). In stroke, TGF-β signaling was increased in microglia and macrophages suggesting that increased TGF-β likely regulated glial scar formation (265). Reports have linked TGF-β or its polymorphisms with atherosclerosis and Alzheimer’s disease (266–268). Other research found TGF-β genotypes associated with longevity in Italian centenarians, a finding not replicated in BELFAST nonagenarians (212, 269). Context-specific environmental factors, epigenetic regulation, and non-coding RNAs are suggested to play a role in TGF-β’s paradoxical pro-and anti-inflammatory functions (7, 270, 271), but important uses have been found for TGF-β in fibrosis management and oncology (272).

#### Interleukin-37

Interleukin-37, formerly an IL-1 cytokine, limits innate inflammation *via* suppression of pro-inflammatory cytokine production (273). Carriage of an IL-37 haplotype that decreases IL-37 levels contributes to increased inflammation. Research demonstrates that IL-37 reduces TNF-α and IL-1β cytokine production from human macrophages, is increased in chronic heart failure patients and attenuated the production of inflammatory cytokines in serum or synovial joints in RA, suggesting IL-37 may have a role in clinical disease (274–276).

## Age-Related Diseases

### Cancer

Cancer increases with aging, with one in two people likely to develop malignant tumors in their lifetime. Probable reasons for this age-related increase include exposure to environmental toxins, declining immune surveillance, and increasingly ineffective DNA repair mechanisms. Inflammation is involved at different stages of tumor development, at initiation, promotion, malignant conversion, invasion, and metastasis, has a paracrine bystander role and is an essential part of the tumor micro-environment. Inflammation also affects immune surveillance and responses to therapy (277). Thus, malignancy is a major threat to successful aging.

While inflammatory pathways are vital to promote immune homeostasis, over-activation or dysregulation can be pathological and lead to malignant progression. Prolonged inflammation, either as a result of chronic infections, or reduced homeostasis in the inflammatory response, plays a role through the production of pro-inflammatory cytokines that may be directly or indirectly implicated in the oncogenesis (278, 279). More recent investigations have focused on the role of the inflammasone pathway, whose biochemical function is to activate casp-1, which leads to the activation of the IL-1β and IL-18 pathways and induction of pyroptosis, a form of cell death. Although inflammasomes have an important role in inhibiting cancer, through the triggering of the programmed-death pathway, they both initiate and maintain carcinogenesis, dependent on tumor type and the tumor environment (280, 281).

Bacterial and viral infections are associated with malignancies. For example, *Helicobacter pylori* (*H. pylori*) infection of the gut is associated with both gastric cancer and mucosa-associated lymphoid tissue (MALT) lymphoma (282). Epstein–Barr virus (EBV) is a causative agent in Hodgkin’s disease (HD), where chronic inflammation is considered a major contributory factor (283), human papilloma virus is implicated in most cases of cervical cancer (284), while human T-lymphotrophic virus 1 (HTLV-1) is a causative agent in adult T-cell leukemia lymphoma (285). A common factor is the association of infection with oncogenesis, with chronic inflammation a contributory factor.

In *H. pylori* chronic infection, elevated levels of IL-1β are detected and recognized as important in the development of gastric carcinoma. Normally gastric acid in the stomach does not permit bacterial survival, but in circumstances of low stomach acidity, *H. pylori* grow vigorously in the mucosa and induces caspase-mediated cleavage of pro-IL-1β and pro-IL-18 in association with the NLRP3 inflammasome. The overexpression of IL-1β induces NF-κB activation and the transcription and expression of IL-6, TNF-α, and IL-10. The proinflammatory cytokine milieu increases the risk for developing both gastric carcinoma and MALT lymphoma (286). Persistently high levels of IL-1β and IL-18 suppress acid secretion, allow hypoacidity in the stomach, loss of parietal cells, gastric atrophy, metaplasia, and eventually gastric cancer. In addition, IL-1β inhibits gastric acid secretion and carriers of IL-1β polymorphisms producing higher IL-1β carry increased gastric cancer risk (287, 288). *H. pylori* infection of gastric mucosa can cause a monoclonal B cell proliferation, with a histological diagnosis of MALT lymphoma. This tumor-like proliferation of gastric mucosal cells and clonal B cells can regress after eradication of the *H. pylori* infection with combined antibiotic therapy and proton pump inhibitor treatment (289).

Viral infections strongly stimulate inflammatory responses and may lead to malignant transformation of the host cell (290). Although the activation of the inflammasome benefits the clearance of viruses and the regression of cancer, there are several examples of viruses, such as EBV and HTLV-1 developing strategies to evade detection, triggering the inflammasome, and high-jacking the inflammatory cascade to induce, and amplify the cancer spread. For example, when EBV infects B-lymphocytes and nasopharyngeal cells through its receptor CD21 (291), this leads to a proliferation of infected B cells, followed by an increase in CD8+ T cells, that controls the infected cells by lysis. However, where the normal infection-limiting response is “exhausted” or dysregulated, B cell proliferation continues unabated leading to chromosomal damage, which drives cell proliferation outside normal control mechanisms and may result in an aggressive non-Hodgkin’s or Burkitt’s lymphoma (292). NLRP3 activation has been demonstrated in EBV-associated cancerous tissues (293). Furthermore, EBV has been shown to be able to overcome the immune response by means of EBV miRNA binding to the 3′-untranslated region of NLRP3 (294), so preventing effective immune activation and control mechanisms.

Retro-viruses stimulate inflammatory responses and are associated with malignant transformation of host cells. They reverse transcribe their RNA into the host cell’s DNA, leading to dysregulation of cellular proliferation and programmed cell death responses, and elicit a pro-inflammatory response. HTLV-1 causes adult T-cell leukemia by targeting CD4+ T cells that express CD25 (IL-2Rα) and FoxP3, similar to Tregs (295, 296). The persistent activation of the NF-κB pathway in HTLV-1-infected T cells and the associated NF-κB oncoprotein Tax contribute to the oncogenic transformation (297). The resulting hijacking of the NF-κB pathway, allows uncontrolled upregulation of cellular genes that govern growth-signal transduction, amplify the pro-inflammatory cytokines (IL-2, IL-6, IL-15, TNF), together with increasing expression of proto-oncogenes (c-Myc), and antiapoptotic proteins (bcl-xl) Hiscott Rayet (298, 299). Inter-individual susceptibility to HTLV-1 infection has been associated with allele carrier status of the NLRP3 gene (300).

In summary, the interaction of infective agents, host cells, adaptive immune cells, cytokine production, and the inflammasome response is complex and incompletely understood. Many cancers arise from sites of infection, chronic irritation, and inflammation, which although sometimes reversible in the pre-malignant phase by eradicating the causative virus or bacterium, often treatments are too delayed to prevent the cancer development. There needs to be improved understanding about the roles of inflammation, the inflammatory cells, and the paracrine effects that allow tumor cell proliferation, survival, and migration. Does the pro-inflammatory environment found in aging enhance and facilitate cancer cell proliferation or does it alternatively represent an upregulated immune surveillance mechanism to deal with increased damaged and dangerous cancer cells? Improved understanding of the pathways involved should begin to provide insights that could contribute to new anticancer and anti-inflammatory therapeutic approaches through manipulation of autophagy for cancer treatment regimes or conversely tagging cancer cells for destruction through proteasome or autophagy upregulation (301).

### Rheumatoid Arthritis

Chronic tissue inflammation has an important role in the etiology and immunopathogenesis of RA (302), with genetic and environmental factors contributing to a predilection to develop the disease. In the *pre-clinical* asymptomatic phase of RA disease, the immune system is characterized by reduced self-tolerance and production of autoantibodies, whereas in the *clinical* phase (303) innate and adaptive immune cells infiltrate the synovial joints and produce symptoms of joint pain and stiffness (304, 305). As RA progresses, immune cells and synovial fibroblasts produce a pro-inflammatory environment in the joint (306, 307) leading to joint destruction (302). Cell-specific cytokines, include TNF-α, IL-1, and IL-6 from macrophages, IL-6, IL-7, and IL-15 from memory T-cells, IL-1 and IL-17 from helper T-cells, and IL-1, IL-6, IL-18, GM-CSF, and TGF-β from synovial fibroblasts (303, 308). This complex cytokine milieu attracts further immune cells, promotes abnormal angiogenesis and osteoclastogenesis, poorly formed leaky vasculature and leads to systemic effects (309).

There is evidence to suggest that activation of the NLRP3-inflammasome contributes to the inflammatory processes in RA. Active RA subjects have increased expression of NLRP3 and NLRP3-mediated IL-18 secretion in whole blood upon stimulation *via* TLR3 and TLR4, but not TLR2 receptors (310, 311). Functional polymorphisms in the genes coding for NLRP3 and its component parts, including CARD8 has been shown to contribute to higher disease activity at diagnosis and for response in the early months of treatment (312, 313).

Patients with RA show premature immune aging and accumulation of CD28^−^ pre-aged effector T cells that associate with disease activation and prognosis (314, 315). A novel T-cell subset CD28^−^ Treg-like cell has been described that produce pro-inflammatory cytokines, mirroring the SASP associated with senescent cells (316). RA patients who show CD28^−^ senescent Treg-like cells in blood seem to demonstrate earlier and more severe osteoporosis (317).

Limiting inflammation before damage occurs is central to successful RA management and the use of specific monoclonal antibodies has been a key therapeutic strategy. The central roles of TNF and IL-6 in RA have been corroborated by clinical trials of biologic drugs, which can specifically target and neutralize these cytokines. Evidence from RA clinical subgroups stratified by responses to specific biologic drugs strongly suggest that for a particular individual, inflammation is coordinated by a predominant cytokine pathway, such as TNF or IL-6 (318).

Anti-TNF biologics, such as adalimumab, etanercept, and infliximab reduce inflammation, pain, neovascularization, lymphocyte infiltration, and increase macrophage apoptosis (318–321). Anti-IL-6R biologics, such as tociluzimab and anti-IL-6, such as sirukumab, strongly reduce disease activity and erosive progression (322, 323). Evidence suggests that the predominant cell cytokines seen in synovial histopathology may act as prognostic biomarkers for stratification of RA patients (324–326).

Studies of TNF and IL-6 gene polymorphisms further support their role in RA risk and severity. SNPs in IL-6 and IL-6R genes associate with increased RA risk and joint damage (327–329), and the TNF 308 G gene polymorphism with RA disease severity and poor response to anti-TNF treatment (330–334). In the elderly person with RA, there is difficulty in distinguishing whether chronic inflammation or genetic “predisposition” initiates disease or if late-onset RA is hastened by the pro-inflammatory phenotype associated with aging. TNF-α inhibitors used as disease-modifying agents in RA improve not only the clinical symptoms of RA, but also decrease the associated vascular risk (335), suggesting that a stratified biologic approach may be of use to therapeutically dampen chronic systemic inflammation related to aging and other age-related diseases.

Like other age-related diseases and aging itself, there is evidence for dysregulation in both the autophagy–lysosomal and the ubiquitin–proteasomal systems in RA (102). Autophagy seems to be activated in RA in a TNFα-dependent manner and regulates osteoclast differentiation and bone resorption, emphasizing a central role for autophagy in joint destruction (336). Gene and allele frequency population differences seem also to contribute to how effectively cellular autophagy processes work within the cell in removing damaged proteins and other necrotic cellular debris. Polymorphisms of the ubiquitn E3 ligase gene that directly influence autophagy have also been identified and have been associated with the etiology and response to drug treatment in RA (337, 338). Both are likely important contributors to the action and effectiveness of disease modifying and monoclonal biological drugs used in RA treatment. The role of the NLPR3 inflammasome may give opportunities for developing other disease-modifying drugs by targeting upstream triggers of the NLPR3 pathway.

### Atherosclerosis

Atherosclerosis is recognized as a chronic inflammatory condition (339) and atherosclerotic plaques show cellular senescence (340, 341). Cytokines are involved in all stages of the pathogenesis of atherosclerosis, having both pro- or anti-atherogenic effects (342, 343). In response to increased low-density lipoprotein (LDL), hypertension, and subsequent shear stress, cytokines modulate endothelial cell permeability and recruit monocytes and T-lymphocytes (344, 345). The continuous monocyte recruitment, foam cell and fatty steak formation eventually result in unstable plaque development, thrombosis, and a cardiac event (345, 346).

Chronic unresolved inflammation is a key feature in atherosclerosis and the levels of SPMs, particularly resolvin D1, and the ratio of SPMs to pro-inflammatory leukotriene B_4_ (LTB_4_), are significantly decreased in the vulnerable plaque regions (27). Vulnerable atherosclerotic plaques are recognized as having distinct features; increased inflammation; oxidative stress; areas of necrosis overlain by a thin protective layer of collagen (fibrous cap). In advanced atherosclerotic plaques, macrophages have more abundant nuclear 5-LOX, which is suggested to lead to conversion of AA to proinflammatory LTs, with the potential to contribute to plaque rupture (27).

The NLRP3 inflammasome, a central regulator of inflammation (58), is activated by cholesterol crystals and oxidized LDL (347, 348) that drives the IL-1β inflammation pathway. Recent research targeting IL-1β inflammation in atherosclerosis using cannakinumab, a therapeutic monoclonal antibody, has shown up to 15% lower rates of recurrent cardiovascular events, which was independent of lipid lowering (349). As well as playing a major role in chronic inflammation, NLRP3 is also upregulated during endothelial cell senescence (350) *via* ROS, and is negatively regulated by autophagy (351, 352). The NLRP3 inflammasome, therefore, appears to warrant further investigation as a potential target for inflamm-aging related to atherosclerosis given that such mechanisms are now of well known importance in atherosclerosis (353).

The gut microbiome has been implicated in age-related inflammation (354) with numerous studies reporting bacterial organisms in arterial plaque (355–357). Emerging research reports bacterial DNA in blood associated with a personal microbiota fingerprint as a predictor of cardiovascular events and stool microbiome as a signature of cardiovascular disease (358, 359). Similarly, bacterial DNA has been noted in cell-free plasma in cardiovascular and chronic renal disease patients (360, 361). Altered gut microbiota composition or dysbiosis is also seen in elderly people, and is associated with inflammatory markers (354). Aging leads to changes in intestinal permeability in gut bacterial milieu (362), and the increased circulatory bacterial DNA observed associated with atherosclerosis support further investigation of the microbiome as a contributory factor to age-related inflammation and atherosclerosis.

### Neuroinflammation and Neurodegenerative Disease

Inflammation has been well established as a major component of neurodegenerative disorders, but it has never been clear if this was a direct cause of the disease or a consequence of the progressive degenerative process that was occurring (363, 364). The central role of cytokines in regulating the immune response has been implicated in neurodegeneration, but over the past decade, there has been a revolution in our understanding of how cytokines contribute to the etiology of the leading neurodegenerative disorders, including Alzheimer’s (AD) and Parkinson’s disease (PD).

In AD, central events seem to include the inflammasome, the NF-κB pathway, and the activation of microglia by a variety of factors, including beta amyloid and pro-inflammatory cytokines (172). Microglia, the primary components of the CNS innate immune system (365), produce cytokines and monitor the integrity of CNS. Together with astrocytes, microglia are the primary effectors of neuroinflammation and express PPRs that allow early recognition of PAMPs and DAMPs. When the NLRP3 inflammasome is activated, the inflammation cascade begins with casp-1 that facilitates the processing of IL-1β and IL18. These proinflammatory cytokines drive the inflammatory cascade through downstream signaling pathways and lead to neuronal damage and death (366). The activated microglia release proinflammatory cytokines, such as IL-1β, IL-6, TNF-α, and IL-18, that contributes to neuronal death and dysfunction.

There is interest in the role of sphingolipid metabolites, such as ceramide and sphingosine-1-phosphate, which regulate a diverse range of cellular processes that are important in immunity, inflammation, and inflammatory diseases (367). Growing evidence suggests that ceramide may play a critical role in NLRP3 inflammasome assembly in neuroinflammation. Research has shown that microglia treated with sodium palmitate (PA) induce *de novo* ceramide synthesis, triggering the expression of NLRP3 inflammasome assembly and resulting in release of IL-1β (368), linking neuroinflammation with dietary lipids. Recent insights into the molecular mechanisms of action of sphingolipid metabolites suggest roles in altering membrane composition, with effects on cellular interactions and signaling pathways with potential causal relationships to neuroinflammatory disease.

Dysregulated autophagy has been considered to play a role in neurodegenerative diseases, particularly AD, and is felt to be a key regulator of Aβ abnormal protein generation and clearance (369). In AD the maturation of autophagolysosomes (i.e., autophagosomes that have undergone fusion with lysosomes) and their clearance are hindered. Evidence suggests that Aβ peptides are released from neurons in an autophagy-dependent manner and that the accumulation of intracellular Aβ plaques is toxic to brain cells leading to AD pathology (370). Furthermore, lysosomal and autophagocytic dysfunction has been associated with both Alzheimer’s and Parkinson’s diseases (71, 72). Senescent cells too, accumulate abnormal protein aggregates in the cytoplasm that contribute to neurodegenerative disease (72). Cellular senescence has been reported in the aging brain with an increase in SASP-expressing senescent cells of non-neurological origin that are likely to contribute to the pro-inflammatory background (103, 371).

In AD and PD, the application of genome-wide association studies (GWAS) has demonstrated a number of key genes, relating to immunity, including the human leukocyte antigen (HLA) complex on chromosome six that regulates the immune and inflammatory response (372, 373). In the most recent Parkinson’s disease GWAS a locus containing the IL-1R2 gene was identified as significantly associated with disease risk and awaits further investigation (372). There is some evidence that carriage of certain pro-inflammatory cytokine gene alleles may confer increased Alzheimer’s disease risk. Single studies have reported that carriers of the A allele of the TNF-α 308 G/A gene were variably associated with increased risk of Alzheimer’s disease (207–210) and that carriage the higher IL-6 producing allele of IL-6 (174 G/C) may confer increased risk (186, 190, 191). Animal studies have provided some clearer understanding of the role of TNF-α in Alzheimer’s disease with evidence of disease modulation with the use of anti-TNF agents (215). Three studies, published in 2013, confirmed a role for the immune response in AD identifying the microglia-related gene TREM2 as harboring an intermediate effect size variant in risk of AD that has also been implicated in other related neurodegenerative diseases (374–376). A recent study of rare variants has also implicated a role for microglial-mediated innate immunity in AD (377).

A better understanding of the molecular pathways involved in the use of established drugs, such as non-steroidal anti-inflammatory or statin drugs in risk and progression of neurological disorders may provide further opportunities to treat earlier or prevent disease onset (378–380). It has been considered that downregulation of the type and magnitude of the pro-inflammatory immune response in neurodegeneration might be a key to earlier and more successful targeting of these pathways. However results, to date, have been disappointing and anti-TNF-α therapies and targeted treatment of TNF-α levels that are elevated in cerebrospinal fluid and in patients’ serum, have produced, at best, modest results (381). Multiple sclerosis patients have benefited from treatment with fingolimod (FTY720) that has been reported to attenuate neuroinflammation, by regulating the activation and neuroprotective effects of microglia, by modulating the sphingosine-1-phosphate receptor (S1P receptor) (382). Given the success of FTY720 for treatment of multiple sclerosis, it is hoped that next-generation S1PR1 modulators will find wider therapeutic uses in other inflammatory disorders. Fingolimod is now under a phase 2 clinical trials for acute stroke and phase 4 for neurodegeneration (383).

## Future Considerations

Aging is heterogeneous among people and highly variable between different organs and tissues. Our genes, our lifestyles, and our response to stress are infinitely individual and variable, so that the immunobiography of each life tells a different story of how each will respond to the internal and external environmental stressors (1–3, 384). But evidence is accumulating that the aging process may be malleable.

Because aging is the major risk factor for age-related diseases, understanding age better and maintaining the health of older people and societies is highly important personally and for societies and governments. Knowledge about the underlying molecular pathways and the genetic and life-style processes associated with age-related disease and aging itself is increasing. Evidence from centenarian and nonagenarian studies suggests that these oldest members of populations have had the ability to delay aging and age-related disease (385, 386). Other studies suggest that centenarians may demonstrate optimized cardiovascular risk factors (387, 388), or have either intuitively or through social example, adopted lifestyles which have interacted with their genes to facilitate a successful aging phenotype (3, 389, 390).

Population studies across the world show that the age-specific incidence of cardiovascular disease, stroke, and dementia is decreasing (391–395). This suggests that better blood pressure and diabetic control and statin use may directly or indirectly link into and downregulate molecular pathways associated with inflammation (396–399). Research into how carriage of certain gene alleles, such as TCF7L2 or IL-6 can increase inflammation or stroke risk, respectively, and can be ameliorated by following a Mediterranean-type diet (42, 400, 401), or how gene splicing and features of senescence may be modulated by resveratrol in food (402), herald research into how gene, diet, and lifestyles can interact, with positive or negative effects on the immune system and health. Increased knowledge is emerging as to how epigenetic modulation can affect cytokine genes with reports linking cytokine epigenetic change to neuroinflammation (403–405). Obesity, smoking, and malnutrition have been shown to have next generational epigenetic effects, and seem likely to contribute to the predilection of offspring developing age-related disease or conversely the longevity phenotype (406–409).

Other strategies should be adopted which link with public health messages and encourage people to adopt behavioral changes in lifestyles. Modifications should include: changes in diets to include more omega-3 containing foods or fruits and vegetables as in the Mediterranean diet (410–413); engagement in regular moderated exercise routines (414–417); continued engagement with social connections and intellectual activities in daily lives (418–420); or best of all a combination of life-style factors (3, 421, 422), all of which have been shown to reduce the inflammatory profile and improve the quality of aging. Although the role of diet on human health and connections through nutrition, inflammation, and cancer are not as linear as those between tobacco, smoking, and lung cancer, obesity is linked to chronic inflammation through several mechanisms, including the dysregulation of autophagy, whereas fasting has anti-inflammatory effects, similar to the effect of exercise (423–426), and may downregulate inflammatory biomarkers (427–429). There is, therefore, considerable interest in the role of the intestinal microbiota and health and the so-called immune-relevant microbiome (324, 354), with important correlations between inflammation and neurodegenerative disease (430), bacterial β-hydroxybutyrate metabolites (431), and the role of vagal stimulation (432).

Increasing evidence shows that many signaling pathways are activated in a stress-type-dependent fashion, and all appear to converge with nuclear factor (NF)-κB signaling, which is a central controller of the immune response, and inflammatory cascade (110, 433–436). With increasing age, immune homeostasis loosens, NF-κB signaling becomes less tightly controlled or is more readily triggered, cytokine dysregulation occurs, and a pro-inflammatory phenotype predominates that underpins most major age-related diseases from atherosclerosis to cancer, and aging itself (Figure 5). Understanding how different factors trigger the NF-κB cascade is an important pathway of research (434). In animal models, miRNA-based regulatory networks involving miR-155 and miR-146a, finely regulate NF-κB activity, with miR-146a downregulating and miR-155 upregulating NF-κB expression (435). There is an important temporal separation of miR-155 and miR-146a cellular expression that allows finely controlled NK-κB signaling and enables a precise macrophage inflammatory response, which merits further research.

Therapeutic opportunities may arise through better understanding of the molecular mechanisms that induce senescent cells and SASP in the cellular environments of chronic disease or whether senescent cells can be removed by upregulating autophagy and using sophisticated tagging mechanisms (110). There will be increased opportunities to use the knowledge gained from clinical studies in autoimmune disease, about the roles and actions of monoclonal antibodies in modulating inflammation, which may be able to be utilized in treatments for other age-related diseases involving inflammation (436). The formulations of new and more specific drugs are likely to become available as the modes of action of kinases, such a AMPK and mTOR which control the senescence and inflammation pathways, become better understood (81, 84, 437). Old drugs, such as metformin, still used in diabetes control, are being repurposed and have been shown to have exciting new uses through their ability to modify epigenetic gene expression. Clinical studies are underway to assess any modulating effect of metformin in aging and age-related diseases (81). The use of histone deacetylating drugs is likely to increase as the clinical use of deacetylation and methylation agents is evaluated in cancer with improved knowledge of their effects and safety criteria (438). The current interest in diet and modified diets will encourage further studies assessing how nutrachemicals modify gene expression, for example, through the regulation of intracellular receptors that bind the promoters of certain genes, and may help to design more specific drugs to modify metabolism and benefit health (439).

Turning research to focus on improved understanding of the mechanisms of inflammation resolution in aging and age-related disease, should also be prioritized, since it is an under researched area. Developing synthetic resolvins for use in inflammation resolution may have advantages over the use of single biological anti-inflammatory blockers in autoimmune disease clinical management, since cytokine networks are highly interactive and complex (440), with many auto-regulatory feedback loops. All these molecular pathways are, or have the potential for being developed as drug targets toward clinical interventions useful in damping down and modulating inflammation (441, 442) and may have a role in delaying the onset or treatment of age-related diseases.

Evidence from on-going global studies of the oldest members of our societies, such as centenarians and nonagenarians (443–454) suggests that it may be possible to delay age-related diseases and that aging may be a potentially modifiable risk factor (455). Further investigation has shown that centenarians and super-centenarians also have an enhanced pro-inflammatory background (9, 456, 457), which at first seems surprising, given their long lives. However, studies have demonstrated that the pro-inflammatory background is accompanied and perhaps modulated, by an enhanced anti-inflammatory status in some centenarians. Some have argued that an enhanced anti-inflammatory phenotype could be beneficial as a contributor to longevity by effectively controlling the pro-inflammatory background (9, 11, 257). Others suggest that some inflammation is good, in the same way as hormetic stress triggers systems, and upgrades them but does not overwhelm them (458). Regular exposure to pro-inflammatory stressors could train the immune system to upregulate and fine-tune its cellular processes, so that it responds better and provides better outcomes, when faced with real life-threatening pathogenic threats.

Genome-wide association studies have proved a powerful methodology to assess the influence of common variation in AD and PD disease susceptibility, but by their nature have reflected low effect size variants that likely have a cumulative effect on risk (459). As next-generation sequencing technology becomes more cost-effective, the ability to identify variants that are less common (<1% minor allele frequency) will become more achievable. These unbiased approaches should aid the identification of key players in the inflamm-aging pathway and will play a critical role in the development of therapeutic intervention strategies in neurodegenerative and age-related diseases.

There is the increasing opportunity to link large global datasets with the technologies of genomics, transcriptomics, and proteomics through bioinformatics and artificial intelligence methods to unlock the physiological, genetic, and molecular pathways that underpin the pro-inflammatory aging-phenotype. Using systems biology methods has the potential to lead to the generation of novel therapeutic approaches for old diseases and modern health challenges. Improving knowledge about how to delay or modify the pro-inflammatory aging-phenotype, the hallmark of aging and age-related disease, will give hope of a better quality aging and the longevity dividend for all.

## Author Contributions

IR conceived and designed the outline of the manuscript. All authors IR, DG, VM, SM, DA, and OR contributed to the manuscript draft. All authors contributed to the drafting and revising of the manuscript and approved the manuscript prior to submission.

## Conflict of Interest Statement

The authors declare that the research was conducted in the absence of any commercial or financial relationships that could be construed as a potential conflict of interest.
